# Vitiligo and atopic dermatitis in young girls: may Koebner phenomenon play a role?^☆^

**DOI:** 10.1016/j.abd.2021.02.014

**Published:** 2022-11-12

**Authors:** Giulia Maria Ravaioli, Annalisa Patrizi, Iria Neri

**Affiliations:** a Dermatology Unit, IRCCS Azienda Ospedaliero, Universitaria di Bologna, Bologna, Italy; b Department of Experimental, Diagnostic and Specialty Medicine Alma Mater Studiorum, University of Bologna, Bologna, Italy

Dear Editor,

Vitiligo is a skin condition in which the pigment melanin is lost from areas of otherwise normal skin. It may be associated with atopic dermatitis (AD) due to the presence of abnormal inflammatory response.^1^

We report two cases of association between AD and vitiligo before 12 years of age.

## Case 1

**Figure 1:**
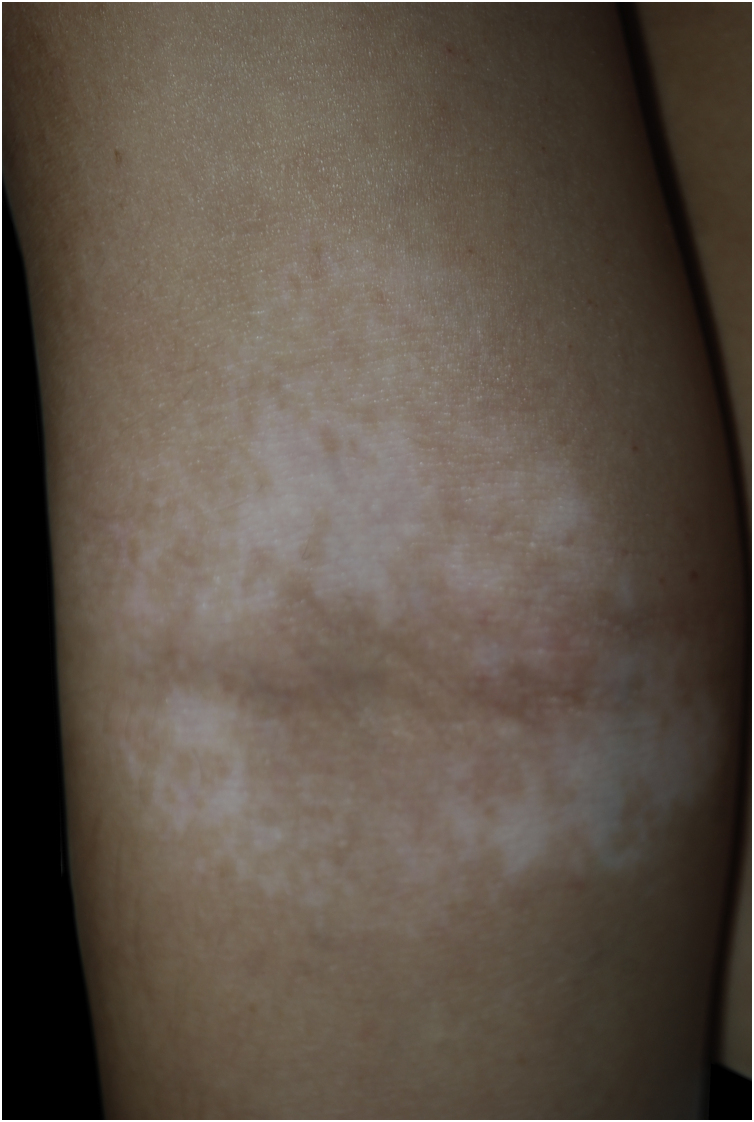
Small hypopigmented macules of the antecubital fossa(Case 1).

**Figure 2:**
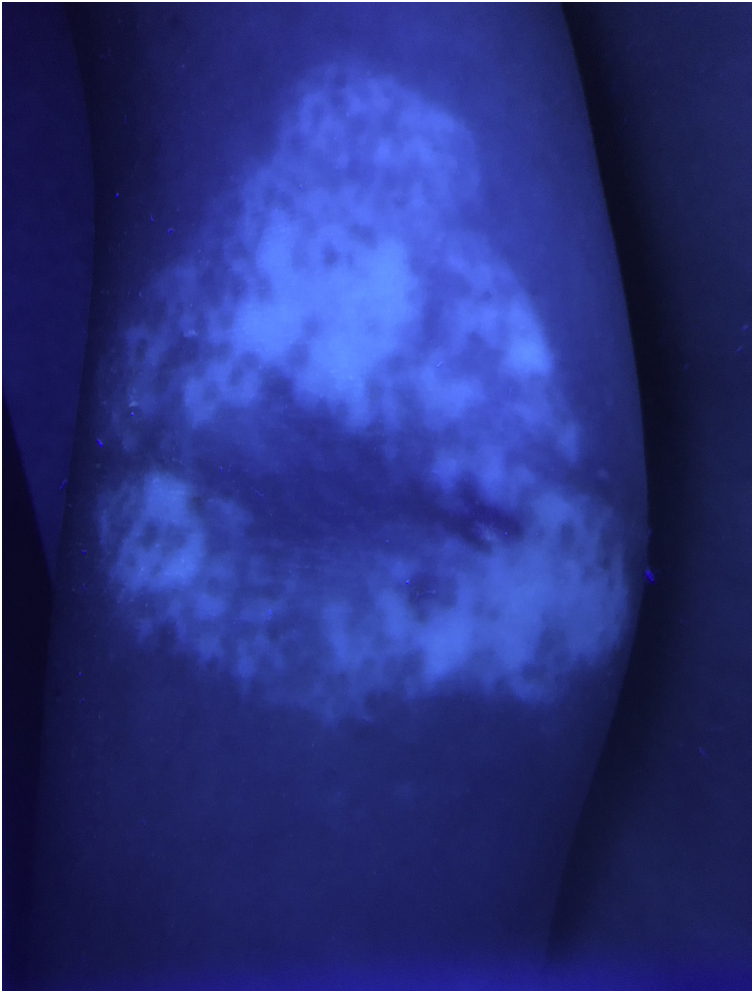
Wood’s lamp examination on hypopigmented macules of the antecubital fossa in Case 1, confirming the diagnosis of vitiligo.

An otherwise healthy 11-year-old girl affected by AD since the age of 2 years, was treated with topical corticosteroids and emollients with good results, later on presenting irregularly hypopigmented areas on the sites that were previously involved by AD (Fig. 1). Similar lesions appeared on other flexural sites and on the face, especially the perioral area. The girl had a familiar history of Hashimoto’s thyroiditis. On clinical examination, the areas were characterized by small macules with clean edges, irregular distribution, and different degrees of hypo- or de-pigmentation. All lesions had appeared after healing of AD lesions, on the same sites. Moreover, no hypopigmented macules occurred outside areas previously affected by AD. Wood’s lamp examination on hypopigmented macules of the antecubital fossae confirmed the diagnosis of vitiligo (Fig. 2).

It showed sharply demarcated emission of bright blue-white fluorescence (Fig. 2), suggesting a diagnosis of vitiligo.

## Case 2

**Figure 3:**
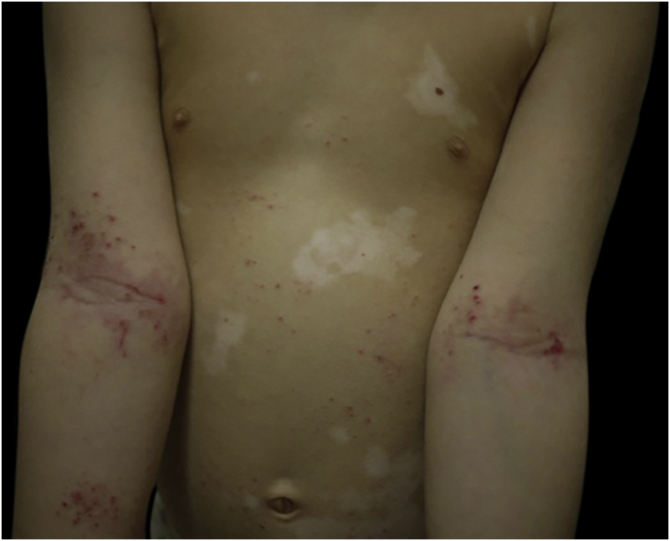
Eczematous lesions affect the typical body areas of AD. Vitiligo areas are different and well-defined.

A 6-year-old girl affected by generalized vitiligo since the age of 4 years, preceded by the appearance of halo nevi, came for consultation due to the onset of eczematous lesions in the typical body areas of AD (Fig. 3). A clinical diagnosis of AD was made. The girl had a family history positive for atopic diseases, in particular rhinoconjunctivitis and asthma.

The two cases present some differences: Case 1 was challenging, since the vitiligo lesions were subtle and hardly visible, and affected the typical AD sites. At first look, they could resemble post-inflammatory hypopigmentation areas. A Wood’s lamp examination was essential for a vitiligo diagnosis. In Case 2, AD and vitiligo affected different body areas, being both easily recognizable. The presence of halo nevi helped the diagnosis.

### Discussion

The association between AD and vitiligo is well-known. A 2015 meta-analysis of 16 studies conducted on adults, observed that early-onset vitiligo was a risk factor for developing AD, compared with late-onset vitiligo.^2^, ^3^

Few studies have focused on vitiligo risk in AD patients: an OR between 1.52 and 1.71 has been reported in young adults and adolescents. In a 2019 meta-analysis, a higher OR was found in adults (4.46) compared with children (2.83).^2^, ^4^

It is not clear if AD more likely precedes or follows vitiligo onset, nor if one of these two diseases triggers the other. No controlled studies have been performed on children younger than 12 years or prepuberal children, where AD is frequent, while autoimmune diseases as vitiligo show an incidence peak around the second and third decades of life.^1^ Vitiligo risk factors are family history of autoimmune disorders, and trigger factors like trauma, skin burns, pregnancy, and systemic diseases. The isomorphic phenomenon of Koebner (PK) was reported in 20%‒60% of vitiligo patients, especially before 12 years of age.^5^

In Case 1, PK may have not directly caused vitiligo but promoted it due to a synergy between the AD pro-inflammatory effect and scratching.

The association between AD and autoimmune diseases has been related to genes/loci of susceptibility influencing the innate immune response and the T cells' function.^1^ AD and vitiligo share some interleukins, they both respond to JAK-STAT pathway inhibitors.^1^ Moreover, an autoimmune mechanism has been hypothesized also in AD: especially during childhood, scratching and barrier dysfunction may favor the entrance of external antigens, which share homologous sequences with human proteins and may induce self-reacting seric IgE.^2^

We believe that, through PK, pediatric AD may preceed the onset of vitiligo in children with risk factors.

## Financial support

None declared.

## Authors' contributions

Giulia Maria Ravaioli: Approval of the final version of the manuscript; critical literature review; data collection, analysis and interpretation; effective participation in research orientation; intellectual participation in propaedeutic and/or therapeutic; management of studied cases; preparation and writing of the manuscript; statistical analysis; study conception and planning.

Annalisa Patrizi: Approval of the final version of the manuscript; critical literature review; effective participation in research orientation; intellectual participation in propaedeutic and/or therapeutic; management of studied cases; manuscript critical review; preparation and writing of the manuscript; statistical analysis; study conception and planning.

Iria Neri: Approval of the final version of the manuscript; critical literature review; data collection, analysis and interpretation; effective participation in research orientation; intellectual participation in propaedeutic and/or therapeutic; management of studied cases; manuscript critical review; statistical analysis.

## Conflicts of interest

None declared.
